# Recommendations for the collection and use of multiplexed functional data for clinical variant interpretation

**DOI:** 10.1186/s13073-019-0698-7

**Published:** 2019-12-20

**Authors:** Hannah Gelman, Jennifer N. Dines, Jonathan Berg, Alice H. Berger, Sarah Brnich, Fuki M. Hisama, Richard G. James, Alan F. Rubin, Jay Shendure, Brian Shirts, Douglas M. Fowler, Lea M. Starita, On behalf of the Brotman Baty Institute Mutational Scanning Working Group

**Affiliations:** 1 Department of Genome Sciences, University of Washington School of Medicine, 15th Avenue NE, Seattle, WA 98195 USA; 2 Current affiliation: Center of Innovation for Veteran-Centered and Value-Driven Care, VA Puget Sound Health Care System, S Columbian Way, Seattle, WA 98108 USA; 3 Division of Medical Genetics, Department of Medicine, University of Washington School of Medicine, Seattle, WA 98195 USA; 4 Current affiliation: Adaptive Biotechnologies, Eastlake Avenue E, Seattle, WA 98102 USA; 5 Department of Genetics, University of North Carolina at Chapel Hill,, Mason Farm Road, Chapel Hill, NC 27514 USA; 6 Human Biology Division, Fred Hutchinson Cancer Research Center, Fairview Avenue, Seattle, WA 98109 USA; 7 Brotman Baty Institute for Precision Medicine, NE Pacific Street, Seattle, WA 98195 USA; 8 Department of Pediatrics, University of Washington School of Medicine, NE Pacific Street, Seattle, WA 98195 USA; 9 Center for Immunity and Immunotherapies, Seattle Children, Research Institute, Ninth Avenue, Seattle, WA 98145 USA; 10 Bioinformatics Division, Walter and Eliza Hall Institute of Medical Research, Royal Parade, Parkville, VIC 3052 Australia; 11 Department of Medical Biology, University of Melbourne, Melbourne, VIC 3010 Australia; 12 Bioinformatics and Cancer Genomics Laboratory, Peter MacCallum Cancer Centre, Grattan Street, Melbourne, VIC 3000 Australia; 13 Howard Hughes Medical Institute, Pacific Street, Seattle, WA 98195 USA; 14 Department of Laboratory Medicine, University of Washington School of Medicine, NE Pacific Street, Seattle, WA 98195 USA; 15 Department of Bioengineering, University of Washington, 15th Avenue NE, Seattle, WA 98195 USA

## Abstract

Variants of uncertain significance represent a massive challenge to medical genetics. Multiplexed functional assays, in which the functional effects of thousands of genomic variants are assessed simultaneously, are increasingly generating data that can be used as additional evidence for or against variant pathogenicity. Such assays have the potential to resolve variants of uncertain significance, thereby increasing the clinical utility of genomic testing. Existing standards from the American College of Medical Genetics and Genomics (ACMG)/Association for Molecular Pathology (AMP) and new guidelines from the Clinical Genome Resource (ClinGen) establish the role of functional data in variant interpretation, but do not address the specific challenges or advantages of using functional data derived from multiplexed assays. Here, we build on these existing guidelines to provide recommendations to experimentalists for the production and reporting of multiplexed functional data and to clinicians for the evaluation and use of such data. By following these recommendations, experimentalists can produce transparent, complete, and well-validated datasets that are primed for clinical uptake. Our recommendations to clinicians and diagnostic labs on how to evaluate the quality of multiplexed functional datasets, and how different datasets could be incorporated into the ACMG/AMP variant-interpretation framework, will hopefully clarify whether and how such data should be used. The recommendations that we provide are designed to enhance the quality and utility of multiplexed functional data, and to promote their judicious use.

## Background

A promise of precision medicine is that genetic information can be used to guide the diagnosis, counseling, and treatment of patients. However, our ability to acquire genetic information has vastly outpaced our ability to understand the relationship between genetic variation and disease. This gap in understanding has led to an explosion in the number of variants of uncertain significance in variant aggregation databases such as ClinVar [1] and BRCA Exchange [2]. Thus, new strategies are needed to aid variant interpretation.

Multiplexed assays of variant effects (MAVEs), in which thousands of sequence variants are assayed for their functional effects simultaneously, can help to address the problem of variant interpretation by providing information about the phenotypic consequences of single nucleotide variants [3–6]. MAVEs have been applied to diverse functional elements, including clinically relevant protein-coding sequences (such as those of *BRCA1* [7–9], *CALM1/2/3* [10], *NUDT15* [11], *PPARG* [12], *PTEN* [13, 14], *SRC* [15], *SUMO1* [10], *TP53* [16–18], *TPK1* [10], and *TPMT* [14]) as well as promoters and enhancers that have been linked to disease [19, 20]. Although these data are being used as evidence to assist variant interpretation in limited cases [1, 21], no detailed recommendations exist that address the specific challenges associated with incorporating multiplexed functional data into variant-interpretation frameworks. This lack of standards has led to confusion about the use of such data and has prevented full realization of their potential benefit.

The American College of Medical Genetics and Genomics (ACMG)/Association for Molecular Pathology (AMP) [22] and, more recently, the Clinical Genome Resource (ClinGen) Sequence Variant Interpretation (SVI) Working Group [23] have developed guidelines for how functional data can be used in clinical variant interpretation. However, these guidelines focus on functional data that are derived from traditional, low-throughput assays, and do not necessarily generalize to multiplexed functional data generated using MAVEs. The comprehensive coverage and quantitative nature of multiplexed functional data mean that rigorous measures of overall data quality and each variant’s functional effect can be calculated. As many, if not all, clinically characterized single nucleotide variants in a gene are assessed in a single MAVE, the functional effect measured for any variant of interest can be viewed in the context of the functional effects of all of the variants in that gene, including the effects of known pathogenic and benign variants. This comprehensive coverage also means that, when compared to assays examining only a few variants, MAVEs are more amenable to rigorous evaluation of assay sensitivity, (i.e., the ability of an assay to identify true-positive pathogenic variants) and specificity (i.e., the ability to avoid false-positive benign variants). If MAVEs are conducted in accordance with a set of coherent recommendations, these metrics of clinical utility, along with other quality and performance measures, can be calculated to assess the suitability of the data as evidence in variant interpretation.

Below, we provide recommendations for the experimentalist seeking to create well-validated multiplexed functional data, and for variant curators who need to evaluate such data for clinical variant-interpretation workflows. In particular, our recommendations are focused on the integration of multiplexed functional data into the ACMG/AMP variant-interpretation framework by establishing a set of principles that can be used to evaluate the strength of evidence (up to PS3/BS3—Pathogenic Strong, Benign Strong) that such data can provide. We begin by addressing the design and reporting of multiplexed functional assays, which are closely related to the issues covered in the new recommendations from the ClinGen SVI working group [24]. We supplement these recommendations with MAVE-specific guidance about how experiments should be conducted and reported, including standards for including internal and external controls. Next, we describe standards for data quality control, including estimation of measurement error. Third, we present recommendations for validation for clinical interpretation, including the assessment of correlation between multiplexed functional data and variants of known clinical effect to determine the strength of the evidence that the data represent. Finally, we present recommendations for using multiplexed functional data as evidence for variant interpretation. As both MAVEs and variant-interpretation workflows are evolving quickly, we end by briefly discussing emerging issues that will eventually need to be addressed in updated recommendations. Data that are collected and described in accordance with our recommendations will be more readily integrated into variant-interpretation workflows, thereby contributing to improved decision-making by patients and providers.

## Methods

These recommendations grew out of discussions among members of the Brotman-Baty Institute’s Mutational Scanning Working Group, a group of researchers and clinicians generating or applying functional data who are based at the University of Washington, the Fred Hutchinson Cancer Research Center, and the Seattle Children’s Research Institute in Seattle, WA. An initial conversation between MAVE developers (Drs. Fowler, Gelman, and Starita) and a molecular genetic pathologist (Dr. Shirts) at the University of Washington led to a proposal to develop standards for the use of MAVE data in clinical workflows. The project was presented at a meeting of the larger working group where the scope and audience were further refined, and the first set of specific recommendations were developed. A summary of the notes from the meeting were circulated, and additional experimentalists (Drs. Berger, James, Rubin, and Shendure) and clinicians (Drs. Dines and Hisama) were included in further conversations to develop the recommendations. Subsequent iterations of the recommendations involved conversations (both in person and via email) among collaborators before agreement on the final content of the recommendations was reached. Dr. Berg and Ms. Brnich are the leaders of the ClinGen SVI Working Group, which developed recommendations for the evaluation of functional data within the ACMG/AMP variant interpretation framework and provided guidance on how to translate those recommendations into the context of multiplexed functional data.

## Design and reporting of multiplexed functional assays

**Fig. 1:**
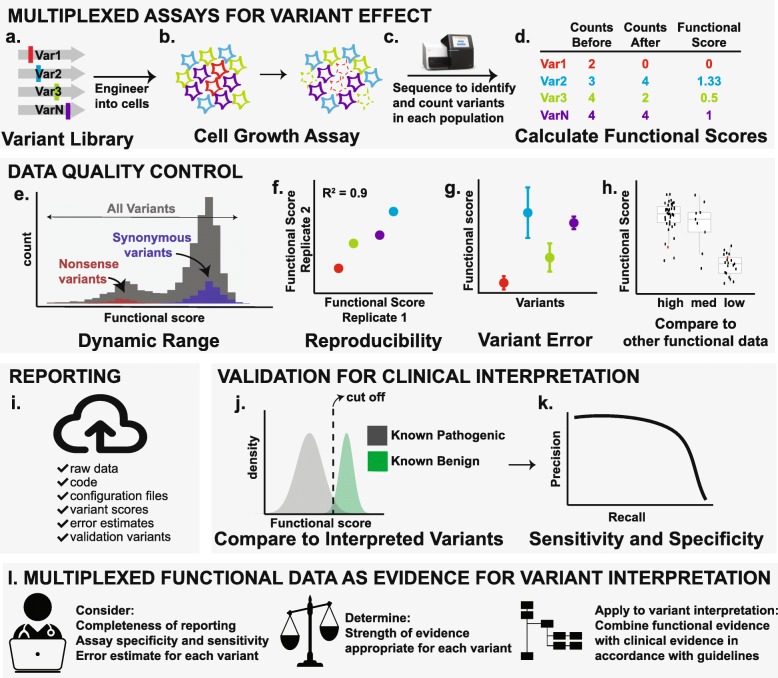
Overview of the steps required to produce, validate and use multiplexed functional data for variant interpretation. Multiplexed assays for variant effect. **a** A DNA variant library is generated and introduced into cells before being **b** subjected to a functional assay. **c** Variants from a sample of each cell population are sequenced and **d** functional scores that reflect their change in frequency are calculated for each variant. Data quality control. **e** The dynamic range of the functional score distribution of the entire library (*gray*) is benchmarked by the observed scores for known functionally normal (*blue*) or abnormal (*red*) variants; here, synonymous and nonsense protein variants are used as an example. **f.** Comparison of functional scores across two or more replicate experiments generates an overall metric of reproducibility (*R*^*2*^). Biological replicates have different input populations that result from separate introductions of the variant library and they provide a better characterization of variation than technical replicates, which use the same starting population. **g** Confidence intervals for each variant functional score are calculated from replicate experiments. **h** Multiplexed functional scores are benchmarked against other measurements of molecular function. Reporting. **i** Sharing of data and analyses facilitates data reuse and enhances data utility. Validation for clinical utility. **j** Functional score ranges are divided into categorical bins. Here, the cutoff between bins is determined by the measured functional score distributions of known interpreted variants. **k** A precision-recall curve is used to assess the assay’s sensitivity and specificity. Multiplexed functional data as evidence for variant interpretation. **l** Clinicians and diagnostic laboratories assess the overall quality of the assay and of specific variant information to determine the weighting, in terms of strength of evidence that should be assigned to the functional information

In a MAVE, a library of DNA variants of a promoter, enhancer, or protein coding sequence is generated through in vitro mutagenesis, DNA synthesis, or by genome editing. Ideally, all possible single nucleotide or codon-altering variants in the target sequence are made (Fig. 1a). The resulting variant library is then subjected en masse to a functional assay in which the variant DNA sequence is directly linked to the readout of the assay (Fig. 1b). For example, in a growth assay, the relative growth of each cell is affected by the functional capacity of the DNA variant within that cell. Alternatively, in a fluorescent reporter assay, each cell becomes fluorescent on the basis of the functional capacity of its variant. The direct linkage of each DNA-encoded variant to cell growth or fluorescence allows changes in each variant’s abundance to be tracked during the functional assay by DNA sequencing (Fig. 1c). Changes in each variant’s frequency in the population before, during, and after selection are transformed into a functional score, which reveals the variant’s effect on function (Fig. 1d). For interested readers, more detail is available in MAVE reviews [3, 6, 25].

Developers of MAVEs who intend that their data should inform variant interpretation should follow the same assay design guidelines as developers of single-variant functional assays [24]. The ClinGen SVI working group encourages assay developers and evaluators to consider the physiological context, meaning how closely the assay reflects the relevant cell or tissue type and its origin. The molecular context, meaning how closely the assay reflects the genetic and functional context of the target gene and associated disease, is also important. However, the SVI group emphasizes that the strength of evidence that an assay can provide should not be based on the design alone, but rather on the predictive power of the assay results. This recommendation is particularly important for MAVEs, which are unlikely to ever mimic all of a gene’s functions perfectly.

Most of the recommendations that we make here focus on how to harness the power of multiplexed functional data to establish the reliability of an assay through careful statistical analysis and validation. The metrics we define can provide high confidence in a multiplexed functional data set even if the assay does not exactly replicate the physiological or molecular context of the disease.

### Multiplexed assay development

#### Assessing assay suitability

A first requirement is that a MAVE must be capable of measuring the disease-relevant effect of variation in the chosen functional element. For example, if gain or loss of function is the primary disease mechanism, then the assay’s dynamic range must be wide enough to capture such changes in function and to distinguish clinically meaningful differences. Thus, to demonstrate assay suitability, either in a pilot phase or in multiplexed form, the dynamic range should be evaluated using variants of known effect (e.g., synonymous and nonsense protein variants). If the measurements for functionally normal and abnormal variants overlap significantly, the assay will not be able to detect changes in function (Fig. 1e). Many genetic diseases are driven primarily by gain or loss of molecular function (i.e., activity). However, other mechanisms, such as changes in expression [26, 27], localization [28–30], or dominant negative effects [18], are also important and must be considered. Moreover, in many cases, the mechanism by which genetic variation causes disease remains partly or wholly unknown. Thus, care must be taken when crafting a MAVE and, in some cases, direct assessment of disease-relevant mechanisms may not be possible.

***Recommendation 1:*** *Assays must have sufficient dynamic range to separate robustly disease-relevant*, *functionally abnormal variant classes such as loss- or gain-of-function from functionally normal variants.*

#### Choosing an assay platform

Experimentalists must ensure that the model system (e.g., cell line or model organism) that they choose is appropriate for the type of variant associated with disease. No model system is appropriate for every application, nor are there a class of models that cannot be used. Instead, the suitability of the chosen model must be assessed relative to the variant type and the disease mechanism. For example, assessing splice variants may require the generation of a variant library by editing the native genomic locus rather than by expressing a mutated transgene. Likewise, the target sequence used to generate the variant library is sometimes optimized for high expression, but if transcript levels contribute to disease, expression level may need to be calibrated carefully to ensure that clinically relevant changes in expression level are not obscured by high baseline levels. Another consideration is that accurate assessment of loss-of-function variants, in particular, may require the endogenous gene to be knocked out or repressed.

To date, most MAVEs have been conducted in model systems such as yeast or in utilitarian human cell lines such as HEK293T, HeLa, or HAP1. Despite early concern about these model systems, variant functional effects that have been measured using them have been highly correlated with variants of known clinical effect. Thus, the gains in predictive accuracy that can be obtained from conducting experiments in more disease-relevant cell lines may be small, especially for genes that are ubiquitously expressed. However, immortalized cell lines that better represent specific cell types or stem-cell-derived differentiated cells may be required for experiments on genes that function in specific contexts or during particular transitions in development.

Even carefully designed assays may be blind to some important functional effects. For example, we recently used a generalizable protein abundance assay to identify possible pathogenic variants of PTEN and TPMT [14]. Known pathogenic active site variants, which damage PTEN enzymatic function but do not affect protein abundance, are not identified by this assay. The inability of this assay to identify all pathogenic variants is not an indication that the assay results are unreliable and unsuitable for clinical use. Nevertheless, a clear explanation of this limitation was essential to ensure that downstream users of the data understand what the assay did and did not measure. Even though a generalizable assay like the protein abundance assay cannot measure all functions, it can still be extremely powerful because a significant portion of damaging variants affect gene expression or stability and, therefore, all protein functions [14, 31]. However, an assay that more completely or specifically represents gene function and disease context may be useful in facilitating a more confident prediction of a benign effect, rather than in predicting pathogenic variants more accurately. Thus, for every MAVE, there should be a clear statement of what was measured and a disclosure of the types of functional effects that were not measured.

***Recommendation 2:*** *Choose an assay design and model system that can assess the type of variant associated with disease. Flag any variants or class of variants that cannot be accurately measured in the released data set.*

### Assay reporting

#### Disclosure of data sets and statistical methods

Given the expense of creating multiplexed functional data sets and their applicability to genomic medicine, it is important that the data sets be Findable, Accessible, Interoperable and Reusable according to the FAIR Principles [32]. These principles ensure that research products are as valuable as possible and are flexible to the needs of the community. Many communities of researchers generating data with clinical applications have highlighted the difficulties that incomplete or inconsistent reporting pose for data re-use [33–35]. Minimum information-reporting requirements [36], data repositories, and shared standards can support the generation and sharing of data that are suitable for reuse.

For MAVE datasets, the primary consideration is that data can be re-used and re-analyzed as the field develops and as more clinically interpreted variants become available. In addition, data from multiple groups must be easily comparable so that conflicting data points are obvious to end users.

Fundamental to meeting all of these requirements is making the underlying raw data, such as sequencing files, read counts, and code for data processing and statistical analysis, easily accessible (Fig. 1d, i). This includes publishing data for each replicate and reporting the configuration files and versions for any published software used in analysis. Raw data availability should be ensured by uploading sequencing data to a public repository such as the Gene Expression Omnibus (GEO) or the Sequence Read Archive (SRA). Analysis files and scripts, including R Markdown and Jupyter notebooks, should be made available on publicly accessible, established platforms such as GitHub or Bitbucket. MaveDB [37] is an open-source database enabling FAIR data sharing for MAVE data. Sequence information, raw data, calculated scores, and experiment metadata are stably associated and can be used for re-analyses, which can also be saved and shared. MaveDB is also accessible via the Application Programming Interface (API), enabling the construction of new tools for data sharing and analysis. Functional scores for variants can also be submitted to ClinVar as supporting evidence [38].

***Recommendation 3:*** *Report data sets using FAIR standards*, *including the reporting of raw data such as sequencing reads and variant counts. Deposit experimental details*, *analytical methods*, *and raw data into applicable publicly accessible databases. To aid transparent and complete reporting*, *we provide a checklist in* Additional file 1: Table S1.

#### Terminology

A universal and precise vocabulary to describe variant effect is important for communicating results. The ENIGMA (Evidence-based Network for the Interpretation of Germline Mutant Alleles) consortium, experts in variant interpretation for hereditary breast and ovarian cancer, developed such a standardized variant effect ontology and it has been adopted by the ClinGen SVI [24, 39]. The ENIGMA terminology avoids classifications such as ‘pathogenic’ and ‘benign’ at the level of functional assay reporting, as these determinations should be made using multiple pieces of evidence and associated with the final variant interpretation. Instead, variants are classified as ‘functionally normal’ or ‘functionally abnormal’, with abnormal variants described further (e.g., loss-of-function, gain-of-function).

***Recommendation 4:*** *To standardize terminology*, *report variant scores using the ENIGMA variant-effect ontology. This will render scores machine readable*, *and will also help to delineate loss- and gain-of-function for end users of the data.*

#### Target sequences

To describe variants scored in a MAVE accurately, it is essential to have unambiguous information about the reference sequence that was used as a basis for the mutagenesis. Many recent studies provide gene symbols instead of stable nucleotide accession numbers [9, 16, 17]. In many cases, this is of little consequence because there are no common minor alleles in the population or alternative transcripts. However, a recent deep mutational scan of TP53 was based on the common p.Pro72Arg allele of TP53 [16], despite the fact that this is not the allele present in the current version of the Reference Sequence database (RefSeq). The p.Pro72Arg allele has been implicated in multiple clinically relevant phenotypes, including cell death rates, and is the subject of hundreds of published papers (see dbSNP entry). Because p.Pro72Arg is so common in the population, this study design is highly relevant for 66% of the population, but the lack of clarity could lead to incorrect interpretation in the cases of patients who harbor the reference allele.

Human Genome Variation Society (HGVS) nomenclature [40] and accurate genomic coordinates will help to identify variants unambiguously. For example, noncoding variants are impossible to communicate without providing exact genomic coordinates. The ClinGen Allele Registry can be used to create or find stable unambiguous Canonical Allele Identifiers (CAID) [41] and provides HGVS nomenclature for all possible transcript and genomic identifiers. To adhere to FAIR principles, experimentalists should avoid creating new formats or redefining existing ones whenever possible.

***Recommendation 5:*** *Include each target’s stable*, *versioned accession number from a common genomic database*, *such as a RefSeq Locus Reference Genome Sequence* [42], *or the full nucleotide sequence*, *either inline in the methods or as a FASTA file in the supplement. Report variants in HGVS nomenclature for nucleotides with a ClinGen Allele Registry identifier.*

## Data quality control

After a MAVE has been performed, the next step is to assess the quality of the multiplexed functional data and to establish the relationship between a reported functional score and the variant’s actual effect on molecular function.

### Replication and error estimates

Using multiplexed functional data for variant interpretation requires an understanding of both the reliability of each variant functional score and the overall reliability of the assay. Therefore, replicate experiments should be conducted to ensure that functional scores are reproducible and to better estimate variant effects.

Early on, replicate experiments were often used as a quality check, evaluating an overall R-squared or similar correlation coefficient (Fig. 1f). These correlation metrics remain useful determinants of overall assay robustness and should be reported. For individual variants, functional scores from different replicates have generally been combined using simple averaging. More recently, statistical frameworks have been developed to incorporate replicate experiments more rigorously, including calculating uncertainties for functional scores for each variant [43]. This gives experimentalists a principled approach to determine the appropriate number of replicates needed to minimize error. Most importantly, calculating individual variant errors gives downstream users valuable information about the trustworthiness of an individual variant score, which may differ from the overall quality of the multiplexed functional data set (Fig. 1g). Although many existing data sets do not report individual variant errors, we strongly encourage experimentalists to take advantage of existing tools and to calculate these errors going forward. If underlying raw data are reported in MaveDB [37] or similar databases, errors can be calculated post-publication.

***Recommendation 6:*** *Conduct replicates to characterize the reliability of assay results. Report correlations between replicates and distinguish them clearly. Error estimates should be calculated for individual variant scores and reported as confidence intervals.*

### Assessment of assay measurements

The reliability of multiplexed functional data should be assessed by testing individual variants that are chosen to span the range of functional scores in a low-throughput version of the same assay. For example, in a growth-based MAVE, the growth rate of variants spanning the functional score range should be tested individually to ensure that the functional score is well correlated with growth rate. In addition, a set of variants will often be chosen for functional assessment in an orthogonal assay to ensure that the relationship between the MAVE-produced functional scores and variant function is well understood (Fig. 1h). For well-studied genes, such as *BRCA1* [8, 9] and *TP53* [16–18], extensive orthogonal validation data sets already exist in the literature and these can be used for comparison. In other cases, orthogonal functional data sets were generated for the purpose of validating MAVE-derived functional scores [12, 14, 15].

Choosing a set of variants to test from across the full range of the functional score distribution is critical for further characterizing the assay’s useful dynamic range beyond looking at the score distributions described above. The set should also include variants with a known effect on molecular function and those with known clinical effects. Sufficient variants should be assessed to allow the calculation of a quantitative measure of consistency between the MAVE functional scores and the validation data (e.g., R^2^, Spearman’s rho). In some cases, it may be that some or all of the variants with known clinical effects tend to have severe functional effects near the extremes of the observed range. If this is the case, consistency in this part of the assay’s dynamic range should be separately assessed because performance at the extremes may be more variable as a result of low sequencing depth (Fig. 1b–d).

***Recommendation 7:*** *Variants from across the full range of assay scores should be tested singly in the same and/or orthogonal functional assay such that a quantitative measure of consistency can be calculated and reported.*

## Validation for clinical interpretation

Before MAVE data can be used for clinical variant interpretation, the relationship between the functional scores and disease association must be confirmed and quantified.

### Curating clinical evidence for MAVE validation

Validation for clinical interpretation requires curating a truth set of interpreted variants, that is, those with an established clinical effect, from literature or from publicly available databases such as ClinVar. Databases such as gnomAD [44], Catalogue of Somatic Mutations in Cancer (COSMIC) [45], OncoKB [46], and the Cancer Genome Atlas [47] are used to determine the allele frequencies of variants in the general population or in tumors. Although there are some exceptions, germline variants that have frequencies greater than 0.5% in the general population are presumed to be benign [48] and can be used for validation [12, 14]. Some MAVEs have also been compared with variants described in linked clinical studies [12].

The number of interpreted variants is typically small relative to the number assessed in a MAVE, so usually all of the interpreted variants that are available are used for validation. Experimentalists should keep in mind, however, that the quality of data from these sources can vary, so they should preferentially use variants whose clinical interpretation is supported by expert panels (such as 3-Star variants in ClinVar). Another consideration is that, to avoid circularity, interpreted variants that have been classified on the strength of functional data should not be used for validation. These considerations are particularly important for the validation of datasets for genes with relatively few interpreted variants, as the inclusion of low-quality or inappropriate data could have an outsized effect on validation metrics. When possible, collaborating with a clinician trained in variant interpretation can help experimentalists to build a robust set of interpreted variants for validation.

***Recommendation 8:*** *List all pathogenic and benign variants chosen for MAVE validation along with their database of origin and*, *if possible*, *their accession numbers or publication references.*

### Determining the predictive value of the multiplexed functional data

The continuous functional scores generated by a MAVE are often binned into categories, such as functionally normal or gain- or loss-of-function, to make them easier to interpret by users. Functional score ranges for the bins can be set in a number of ways: an arbitrary cut-off can be used, or the categories can be defined by significant overlap with variants of known molecular function [8, 14] or clinical effect [10, 12] (Fig. 1j). Then, the predictive value of these variant categorizations is evaluated by constructing a receiver operating characteristic (ROC) or precision-recall curve using the curated set of validation variants (Fig. 1k). Sensitivity and specificity values that are based on these categories are the standard metrics for conveying the clinical utility of a MAVE. Although they have yet to be used in the context of multiplexed functional data, positive and negative predictive values may also be calculated and have the potential to be more informative because they will account for the presumably low prevalence of pathogenic variants in many genes. If all of the underlying data are appropriately reported, end users can calculate the performance metrics that are most appropriate for their needs.

Even if a MAVE does not perform well according to some statistical measures of clinical utility, it may still provide valuable information for variant interpretation. For example, results from a high-specificity assay may be used to ‘rule-out’ a variant as being abnormal for that function even if the sensitivity is modest. In this situation, the multiplexed functional data set may be very useful in providing evidence for classifying benign variants, but not as useful for classifying pathogenic variants. The opposite may be true of a data set with high sensitivity and modest specificity. We note that standard cutoffs for acceptable assay performance (e.g., sensitivity and specificity > 90%) are based on experience with targeted diagnostic sequencing tests, where a single gene or small panel of genes are sequenced or assessed for a single variant of interest in patients in whom the probability that a variant is causative is relatively high. In a screening context, such as whole genome or exome sequencing, however, there is a low probability that any single detected variant is causative. The appropriate predictive thresholds for such applications will differ from those that are currently established, and MAVE standards will need to evolve with clinical standards for this workflow [49].

We note that the level of validation that is feasible depends on the number of known pathogenic and benign variants, so an iterative process should be established to allow the updating of validation metrics as more interpreted variants become available, enhancing the lifetime and applicability of the multiplexed functional data.

MAVEs can be designed to assess a spectrum of mild to severe phenotypes that are associated with variants in a single gene. For example, in a MAVE that measured the phosphatase activity of PTEN variants, variants that were associated with autism spectrum disorder had less severe functional effects than those associated with PTEN hamartoma tumor syndrome [13]. For MAVEs that are designed to assess variants associated with multiple clinical phenotypes, each variant should be validated separately.

***Recommendation 9:*** *Report the predictive value of multiplexed functional data in terms of sensitivity and specificity. Validate and report each gene–disease pair separately.*

## Multiplexed functional data as evidence for variant interpretation

If the above recommendations for data production and reporting have been followed, and a trustworthy and transparent multiplexed functional data set has been produced, the data can be incorporated with existing clinical and in silico evidence for or against pathogenicity.

### Germline variants

#### Incorporation into the ACMG/AMP variant-interpretation framework

Multiplexed functional data that meet the above requirements can be incorporated into the ClinGen SVI framework [24] for variant interpretation, where the strength of evidence that can be applied for a specific variant (up to the ACMG/AMP PS3/BS3) will be determined by the overall quality of the assay, the quality of the data reporting, the assay’s predictive power, and the individual variant score and its confidence interval (Fig. 1l). Multiplexed functional data that do not meet the above recommendations for assay design, quality control, reporting, and validation should be used with caution and, in general, their evidence strength should be capped at the supporting evidence level (see decision tree in Additional file 1: Figure S1). As a multiplexed functional dataset is re-evaluated with newly interpreted validation variants, the strength of evidence provided by that dataset may change.

Clinical workflows are evolving towards quantitative variant assessment, and multiplexed functional data can also be incorporated into these new workflows. Recently, Bayesian odds of pathogenicity (OddsPath)—corresponding to the ACMG/AMP criterion for supporting, moderate or strong evidence—have been determined [50], and clinicians and variant curators are encouraged to move towards the use of more quantitative assessments of variant pathogenicity. An OddsPath can be calculated for multiplexed functional data and incorporated directly into a Bayesian framework [24] or equated to a supporting, moderate, or strong evidence level [50] depending on the individual variant’s functional score and error estimate.

***Recommendation 10:*** *The strength of evidence that can be provided by the multiplexed functional data should be determined on a variant-by-variant basis that accounts for both the error associated with the measurements for the specific variant and the overall trustworthiness and predictive power of the assay. Once an evidence level is determined*, *it can be incorporated into the ClinGen SVI and ACMG/AMP variant classification frameworks like any other functional assay data.*

#### Evidence stacking

We recommend that functional assays should only be used once as a pathogenic supporting, moderate, or strong criterion. This is because, in general, it will be nearly impossible to determine whether the separate MAVEs are measuring orthogonal or overlapping functions. Concordance between two functional assays for a variant does add reassurance that functional scores are robust. Machine-learning models can be used to incorporate functional scores from several different assays that were applied to the same functional element to predict an overall variant effect. Initial efforts combining data from multiple MAVEs that query different functions of the same protein have shown that the prediction of pathogenic variants can be improved by integrating the datasets [7, 11, 16]. The improvements in predictive performance are likely to come from the formation of a more complete picture of variant effect. These composite scores should only be used once and should not be stacked with scores derived from individual functional assays, or from orthogonal assays that are not included in the composite score.

***Recommendation*** 11***:*** *Do not stack evidence from multiple MAVEs for the same variant. If multiplexed functional data for the same variant are conflicting*, *use the results of the most well-validated assay or do not use functional data as evidence. A possible exception is separate splicing and protein functional data that both point to a variant being functionally normal*, *which may be used as independent pieces of evidence in support of a benign interpretation.*

### Somatic variants

Variant interpretation is increasingly a standard part of clinical oncology, especially in tumor types where genotype-driven therapies are standard-of-care. The AMP/American Society of Clinical Oncology (ASCO) guidelines for somatic variant interpretation focus on the therapeutic and diagnostic or prognostic significance of the variant [51]. Somatic variants of strong clinical significance, Tier I, can be used to predict response or resistance to US Federal Drug Administration (FDA)-approved therapies, both on- (Level A) or off-label (Level B). Tier II variants, which are of potential clinical significance, can be used as inclusion criteria for clinical trials of investigational treatments. Tier III variants are variants of uncertain (or unknown) significance (VUS) and should not be used alone to determine clinical significance. Tier IV variants have no evidence that suggests a role in cancer and are considered benign. The AMP/ASCO guidelines do not specifically define a role for functional data, although they can be used as evidence supporting Tier I, II, and IV classifications.

Although specific guidelines for the use of functional evidence for somatic variant interpretation have yet to be written, one could imagine that prospective maps of all possible resistance mutations in a drug target would provide valuable information, or that the identification of all possible loss-of-function variants in tumor suppressors would help to inform the interpretation of both somatic and germline variants. In addition, the bar for including functional data as evidence for somatic-variant interpretation may be lower than it is for germline variants, as the patients are already under treatment for a known disease. Nevertheless, assay quality, reporting, and validation requirements should not be loosened for somatic targets. FAIR result reporting is possibly more important for somatic variants than for germline variants: as more tumor sequencing is conducted, validation metrics can be updated in the light of post-publication re-analysis of multiplexed functional data.

***Recommendation 12:*** *MAVEs directed at somatic variants should follow all of the above assay design*, *data quality*, *and reporting guidelines. As interpretation standards for somatic variants evolve*, *and an increasing number of clinically relevant somatic variants are reported*, *datasets following these standards will be well-positioned for re-analysis.*

## Future directions

The development of MAVE methods is a fast-moving field. In the near future, we envision vast improvements in machine-learning methods to predict functional effects for unseen variants and in new assays to gain access to additional cellular phenotypes.

### Machine learning models for imputation

The large amounts of data generated by a MAVE are a natural starting point for machine-learning models that can predict missing functional scores, or that could even be applied to genes without multiplexed functional data. Indeed, multiplexed functional scores—along with structural information, physiochemical properties, and evolutionary information—have already been used to impute scores for variants that were not measured in the assay [10, 52]. These imputed scores lie somewhere between empirical functional scores and in silico data, which are limited to being used as supporting evidence in the ACMG/AMP framework.

The performance of these predictive models is promising, and in some cases, the correlations between the imputed scores and known functional effects are similar to correlations with measured scores. Eventually, confidence in imputed scores could be high enough that they could be used as strong evidence for variant interpretation. Currently, however, imputed scores should be kept separate from measured scores and should be used similarly to other in silico data (i.e., the strength of evidence should be capped at the supporting level). New standards will be needed to address how the results of such models for missing data can be incorporated into cohesive variant classification workflows.

### The next generation of MAVEs

To date, MAVEs have queried simple cellular phenotypes such as gene expression, cell growth, or reporter activation. Although fruitful, these first efforts ignore many complex cellular phenotypes that are currently impossible to interrogate in a multiplex fashion. The current assays are not informative in deciphering variant-specific effects on a cell’s biology. For example, we might learn that a variant is required for cell survival or reporter activation, but it would be more valuable to understand whether the cell remodels signaling pathways when expressing a variant, or whether it upregulates a gene that could be targeted by a drug or that causes a change in the differentiated state of the cell. The next generation of MAVEs will allow access to more phenotypes, such as cell morphology, behavior, differentiation, and transcriptional state. The increased information content generated by these new assays will require new statistical frameworks to relate these more complex readouts to disease relevance.

## Conclusions

There is a noticeable gap between the large amount of data being generated by MAVEs and the small amount that has been used for the clinical interpretation of variants. We brought experimentalists and clinical geneticists together to identify barriers that were precluding the use of multiplexed functional data and to develop a set of recommendations to help close the gap.

First, we determined that experimentalists were not validating and communicating their data sets in a way that led to the transparency and rigor required for clinical uptake. We addressed these shortcomings with recommendations for assay design and reporting (Recommendations 1–5), data quality control (Recommendations 6 and 7), and validation for clinical interpretation (Recommendations 8 and 9). In addition, we supply a checklist in Additional file 1: Figure S1 for experimentalists to use as a template to ensure that their multiplexed functional data are thoroughly reported, tightly controlled, and well validated. Our hope is that completed checklists will also serve as a document that variant curators use to assess adherence to our recommendations and the clinical utility of their data, thus making the data easier to use.

Second, like other groups, we found that there was confusion regarding the application of the ACMG/AMP’s PS3/BS3 criterion for using ‘well-validated functional data’ in variant interpretation frameworks. The ClinGen SVI Working Groups’ updated recommendations [24] provide a much clearer picture of what a ‘well-validated assay’ actually looks like and, importantly, communicate that not all functional data will reach the level of strong evidence. Here, we expanded upon those recommendations as they pertain to multiplexed functional data (Recommendations 9–11). To aid this process, we also provide a decision tree in Additional file 1: Figure S1 so that clinicians and variant curators can use assay-level (provided in Additional file 1: Table S1) and variant-level (provided by the variant score and/or categorization) information to determine the appropriate strength of evidence provided by multiplexed functional data for variant interpretation. Our hope is that by combining the two sets of recommendations, multiplexed functional datasets will be more readily incorporated into the ACMG/AMP variant-interpretation framework.

An explosion in clinical genetic sequencing is resulting in a rapid increase in observed genetic variation, but with a much smaller increase in variants that have a known impact on patient health, disease progression, or treatment. The generation of multiplexed functional data for disease-relevant genes provides a way forward—evidence for a clinical interpretation of newly observed or rare variants can be provided by prospective evaluation of a variant’s effect on gene function. Our recommendations seek to bridge the gap between multiplexed functional data and existing clinical variant-interpretation frameworks, so that multiplexed functional data can be combined with other sources of evidence to more rapidly assign a meaningful clinical interpretation to a larger number of observed variants.

## Supplementary information

**Additional file 1: Table S1.** A checklist for experimentalists to create complete data sets. **Figure S1.** A decision tree for using multiplexed functional data in clinical variant interpretation.
