# Supplementary material for: Recommendations for the collection and use of multiplexed functional data for clinical variant interpretation
Source: Genome Med. 2019 Dec 20;11:85. doi: 10.1186/s13073-019-0698-7 (PMC6925490; doi:10.1186/s13073-019-0698-7)

Supplementary Table 1 - Recommendation Checklist for Experimentalists

This summary table of our recommendations can be incorporated into the supplementary information of published MAVE datasets - giving experimentalists a structured way to track their adherence to the recommendations, thus helping clinicians and variant curators to evaluate the datasets.

**Recommendation**	**Fulfilled? (Y/N)**	**Additional information (performance metrics, links, etc)**
Target Sequence or Accession		
Disease of interest	*Disease targeted for variant interpretation using MAVE data*	
Variant type assayed	*e.g. protein coding variants, splice variants, etc*	
Are any variant types inconclusively assayed	*e.g. active site variants*	
Data and experimental details are shared in publicly accessible databases and repositories		
- Sequence reads	Y/N	*URL or link*
- Functional scores	Y/N	*URL or link*
- Analysis files or scripts	Y/N	*URL or link*
Variants categorized using ENIGMA ontology	Y/N	
Functional scores reported with variants in HGVS nucleotide format	Y/N	
Quantitative measure of MAVE functional score concordance with external validation data calculated	Y/N	*Measure calculated and value*
Replicates conducted	Y/N	*Type of replicate (biological or technical) and number*
Individual variant confidence	Y/N	

intervals calculated and reported		
Interpreted variants used for assessment of clinical utility provided in HGVS nucleotide format with source database	Y/N	*Insert rows below with validation variant HGVS sequences and source database or provide link to list in stably accessible repository*
Sensitivity calculated	Y/N	*Calculated value, add rows if more than 1 disease validated*
Specificity calculated	Y/N	*Calculated value, add rows if more than 1 disease validated*

Supplementary Figure 1 - Decision tree for clinicians and variant curators evaluating multiplexed functional data.

Multiplexed functional data meeting these recommendations can be incorporated into the ACMG/AMP variant interpretation workflow. Clinicians and variant curators can use assay-level information provided in a completed version of Supplementary Table 1 to determine the maximum strength of evidence, and variant-level information provided by the variant score and/or categorization to determine the functional effect. Scores that su pport use in clinical variant interpretation are further evaluated by their consistency with other available functional data (only one piece of functional data should be used as evid ence). Assessing the variant scores in a quantitative framework, such as OddsPath, can increase the strength of evidence that can be applied for variant interpretation.

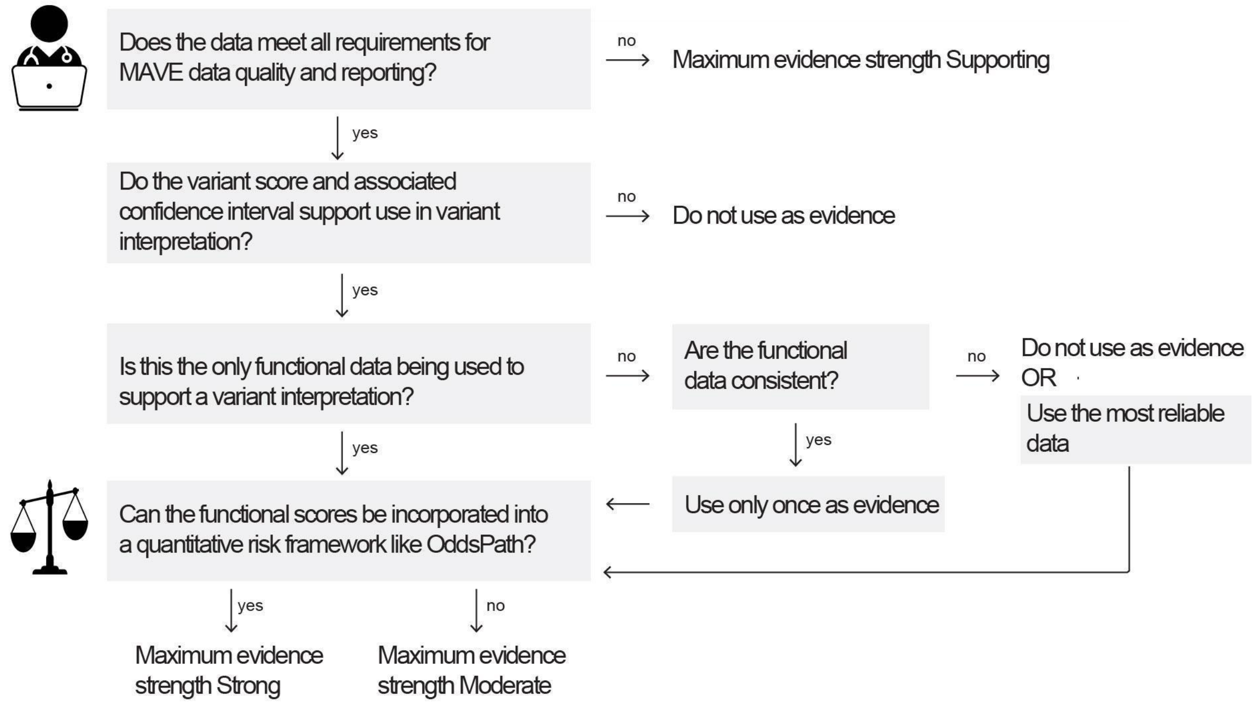

Supplement: Supplementary file 1 — Additional file 1: Table S1. A checklist for experimentalists to create complete data sets. Figure S1. A decision tree for using multiplexed functional data in clinical variant interpretation. [file 13073_2019_698_MOESM1_ESM.pdf]
